# Antiviral prophylaxis during pandemic influenza may increase drug resistance

**DOI:** 10.1186/1471-2334-9-4

**Published:** 2009-01-20

**Authors:** Martin Eichner, Markus Schwehm, Hans-Peter Duerr, Mark Witschi, Daniel Koch, Stefan O Brockmann, Beatriz Vidondo

**Affiliations:** 1 Department of Medical Biometry, University of Tübingen, Tübingen, Germany; 2 ExploSYS GmbH, Leinfelden-Echterdingen, Germany; 3 Division of Communicable Diseases, (SFOPH) Swiss Federal Office of Public Health, Bern, Switzerland; 4 Department of Epidemiology and Health Reporting, Baden-Württemberg State Health Office, District Government Stuttgart, Germany

## Abstract

**Background:**

Neuraminidase inhibitors (NI) and social distancing play a major role in plans to mitigate future influenza pandemics.

**Methods:**

Using the freely available program *InfluSim*, the authors examine to what extent NI-treatment and prophylaxis promote the occurrence and transmission of a NI resistant strain.

**Results:**

Under a basic reproduction number of R_0 _= 2.5, a NI resistant strain can only spread if its transmissibility (fitness) is at least 40% of the fitness of the drug-sensitive strain. Although NI drug resistance may emerge in treated patients in such a late state of their disease that passing on the newly developed resistant viruses is unlikely, resistant strains quickly become highly prevalent in the population if their fitness is high. Antiviral prophylaxis further increases the pressure on the drug-sensitive strain and favors the spread of resistant infections. The authors show scenarios where pre-exposure antiviral prophylaxis even increases the number of influenza cases and deaths.

**Conclusion:**

If the fitness of a NI resistant pandemic strain is high, any use of prophylaxis may increase the number of hospitalizations and deaths in the population. The use of neuraminidase inhibitors should be restricted to the treatment of cases whereas prophylaxis should be reduced to an absolute minimum in that case.

## Background

Neglecting the possible emergence of NI resistance, modeling studies have suggested that an influenza pandemic may be contained if treatment and prophylaxis are introduced immediately [1-3]. Switzerland has stockpiled sufficient NI to treat 25% of the population and considers using some of the stockpile for prophylaxis in health care workers and essential services (fire brigade, police, etc). As influenza viruses mutate constantly, widespread use of antivirals in the case of a pandemic could cause selection pressure which could lead to the emergence and spread of NI resistant strains. *De novo *emergence of a resistant strain does not necessarily cause negative outcomes; it is the transmission fitness of the resistant strain which plays a major role [4-7]. Simulation studies on HIV [8] and HSV-2 [9] have also shown the important role of transmissibility in driving the dynamics of drug resistance. Influenza resistant strains that show nearly the same transmission fitness as wild type strains have been detected [10]. Also, data from the European system VIRGIL show, that a NI resistant seasonal influenza A (H1N1) strain circulates in several countries [11]. NI resistance leads to treatment failures and thus, to an increased number of hospitalizations and deaths attributable to influenza. As antiviral prophylaxis and treatment are relevant for future interventions against influenza, understanding the implications of NI resistance and the dynamics of its spread is essential. We examine how NI treatment and prophylaxis contribute to the emergence and circulation of NI resistant strains and to changes in the epidemiology of infection.

## Methods

We extend the freely available deterministic simulation program *InfluSim *by including the emergence and the spread of NI resistance [12]. *InfluSim *version 2.2 distinguishes drug sensitive and resistant infections, and allows for prophylaxis [13].

**Figure 1:**
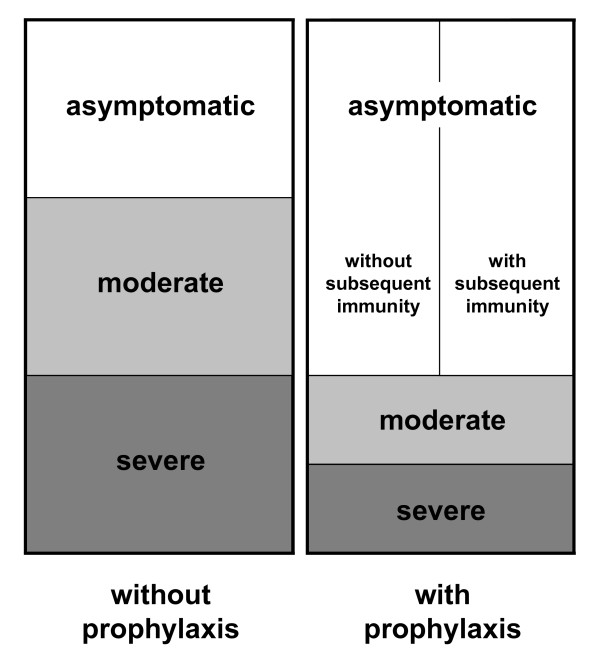
**Course of disease**. Model assumptions on the course of disease of cases with and without prophylaxis if people are infected with the drug sensitive virus. Without prophylaxis, one third of infected individuals remains asymptomatic, one third becomes moderately sick and one third becomes severely sick and needs medical help. Prophylaxis halves the fractions of moderately and severely sick individuals and doubles the fraction of infected individuals who remain asymptomatic, but only half of the asymptomatic infections lead to protective immunity.

We employ a basic reproduction number of *R*_0 _= 2.5 [14], and assume that one third of all infected individuals remain asymptomatic, that another third becomes moderately sick and that the remaining third becomes severely sick and seeks medical help (Fig. 1). A proportion of severely sick cases needs hospitalization and may die from the disease, depending on the age and on the risk group of the patient (see Additional file 1, Table A1). Adults who develop severe disease are sick and contagious for 7 days on average and need 5 more days to recover before they can resume work. Treatment reduces their remaining duration of the disease by 25%, their contagiousness by 80% [2] and their need of hospitalization (and concurrently their risk of death) by 50% [15]. A fraction of the population between 20 and 60 years of age is offered antiviral prophylaxis. We assume that people who take prophylaxis are less susceptible to the infection with the drug sensitive strain (the standard value of 50% is varied in an uncertainty analysis). If they are infected, twice as many of them remain asymptomatic than without prophylaxis; their contagiousness, their duration of being sick and their need for hospitalization are reduced in the same way as described above for therapeutic treatment. We furthermore assume that only half of the individuals who take prophylaxis become immune after asymptomatic infection whereas the other half is left susceptible (Fig. 1).

Simulations start with a single drug sensitive infection in a Swiss population of 100,000 inhabitants. We assume that 4.1% of children up to 12 years of age and 0.32% of teenagers and adults infected with the drug sensitive virus develop a NI resistant infection if they take antiviral drugs [16,17]. The probability that they pass on the resistant virus to others is small, as on average their contagiousness has already decreased considerably before the resistant virus appears. This is because (i) patients seek medical treatment on average 24 hours after onset of symptoms, (ii) their contagiousness is assumed to decline exponentially over time with 90% of the total infectiousness being spent within the first half of the infectious period, and (iii) if *de novo *NI resistance occurs in a patient, it occurs at a random time point between treatment and the end of contagiousness. The resistant virus can have a reduced transmissibility (fitness) compared to the drug-sensitive strain, while having the same pathogenicity, causing the same natural history of disease and inducing the same degree of immunity, but cases infected with the NI resistant virus no longer respond to antiviral drugs. Simulations are performed for a variety of parameter values: The fitness of the NI resistant strain is varied from 80 to 100%. The percentage of adults between 20 and 60 years of age who receive prophylaxis is varied from 0 to 20%. Prophylaxis is assumed to reduce the susceptibility of the recipient by 50% (varied in an uncertainty analysis from 0 to 100%). Fifty percent of prophylactically treated individuals who experience asymptomatic infection become immune after clearing the infection (varied in an uncertainty analysis from 0 to 100%). We run simulations with and without prophylaxis, with and without resistance, and observe the total number of hospitalizations due to severe influenza and the total work loss for people who receive prophylaxis. No ethics committee was required to grant permission for this investigation.

## Results

### Simulation results

**Figure 2:**
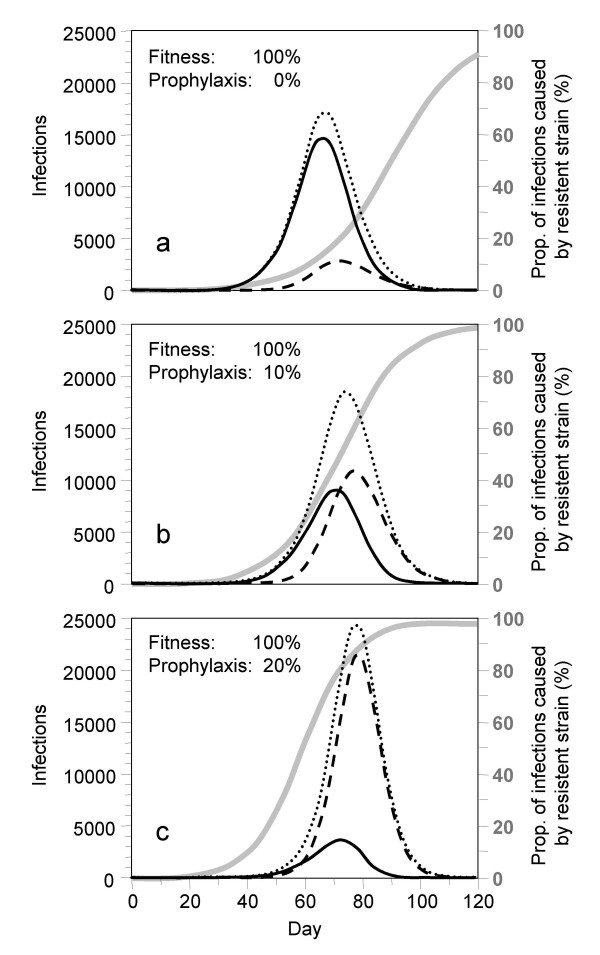
**Prevalence of infection**. Prevalence of people infected with the drug sensitive virus (solid lines), the drug resistant one (dashed lines) and the sum of both (dotted lines). All cases who seek medical help receive antiviral treatment; additionally, a fraction of (a) 0%, (b) 10% and (c) 20% of all adults between 20 and 60 years of age are given prophylaxis. The grey curves and the right hand scales indicate the fractions of resistant infections among all infections. Assumptions: (1) A single drug-sensitive infection is introduced on day 0 into a Swiss population of 100,000 individuals. (2) Resistance develops *de novo *in 4.1% of children and 0.32% of adults who receive medication. (3) Social distancing reduces the number of contacts by 10% for all individuals; isolation additionally prevents 10%, 20% and 30% of contacts of moderately sick cases, severely sick cases at home, and hospitalized cases, respectively. (4) Antiviral treatment reduces the contagiousness of patients by 80%, their duration of sickness by 25% and their need of hospitalization by 50% if they are infected with the drug sensitive virus. (5) Prophylaxis furthermore reduces susceptibility by 50%. Upon infection, it doubles the fraction of individuals who stay asymptomatic from one third to two thirds, but only one of the two thirds becomes immune. (6) *R*_0 _= 2.5 for the drug sensitive and the drug resistant virus (fitness = 100%).

Figures 2a–c show how the prevalence of infection with the drug sensitive and the resistant virus change during the pandemic wave if all severe cases are treated with NI and if additionally 0%, 10% or 20% of the people between 20 and 60 years of age receive prophylaxis (assuming that the fitness of the NI resistant strain is as high as that of the drug-sensitive one, i.e. 100%). The simulations start with one drug-sensitive infection in a susceptible population of 100,000 individuals. NI resistance develops *de novo *and gradually builds up during the epidemic wave. If no prophylaxis is given, the drug-sensitive strain dominates most of the epidemic wave (full curve) and the resistant strain (dashed curve) only becomes prevalent in the end (Fig. 2a).

Prophylaxis increases the pressure on the drug sensitive strain and favors the transmission of the resistant strain. The prevalence of the NI resistant strain increases considerably if 10% of all people between 20 and 60 years of age are given prophylaxis (Fig. 2b). If the prophylaxis coverage is increased to 20%, the resistant strain very quickly dominates the epidemic wave and the majority of cases are infected with the resistant strain (Fig. 2c). With increasing prophylaxis coverage, the peak number of infected individuals increases from 17,000 to 25,000 and the total number of infected individuals increases from 62,000 to 72,000 (Figs. 2a–c).

Individuals who are infected with the NI resistant strain do not respond to antiviral treatment. Even without any prophylaxis in the population, 13.7% of all treated patients are treated in vain due to resistant infection. This fraction increases to 43.4% and 74.5%, respectively, if 10% or 20% receive prophylaxis.

**Figure 3:**
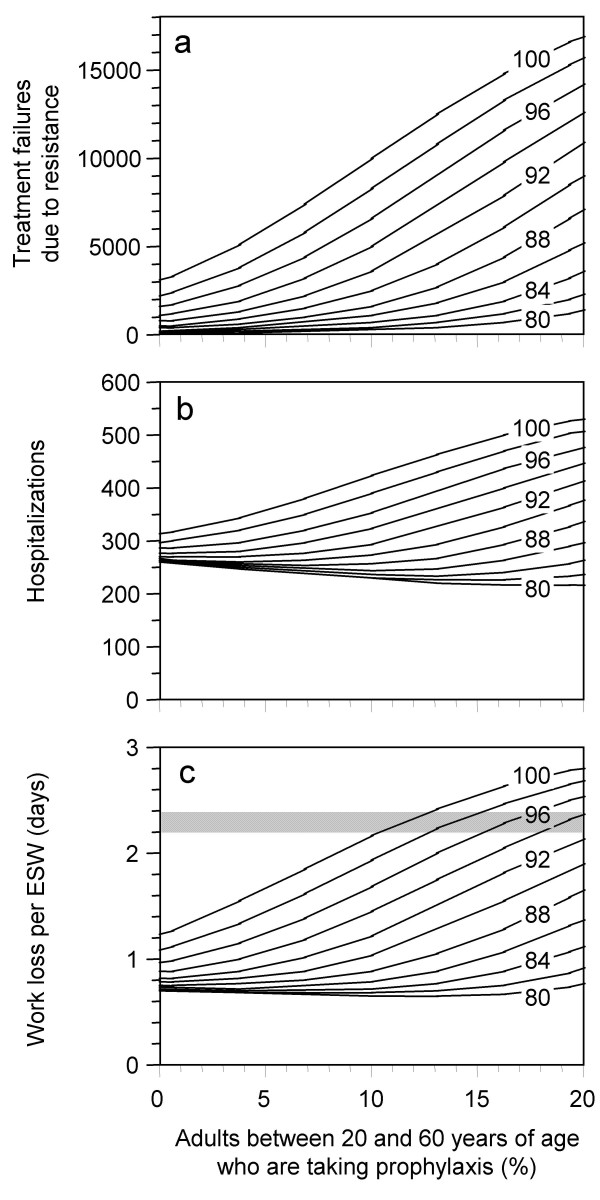
**Influence of antiviral prophylaxis**. Influence of antiviral prophylaxis and of the fitness of the resistant strain on a pandemic wave in a population of 100,000 individuals into which a single nonresistant infection is introduced on day 0 (development of resistance and other details see Fig. 2 and text). The horizontal axis shows what percentage of the population between 20 and 60 years of age receives prophylaxis; the numbers 80 ... 100 in the graphs indicate the fitness of the resistant strain. Simulations with lower fitness values lead to curves which are nearly identical to the 80% fitness curves. The results are given as (a) total number of treatment failures due to drug resistance; (b) total number of hospitalizations; (c) expected duration of work loss per ESW (essential service worker), i.e. per person who receives prophylaxis. Even without any prophylaxis in the population, the average work loss per person is slightly modified by the fitness of circulating resistant virus (because of treatment in the population). The grey horizontal bar shows the range of work loss per person which must be expected without prophylaxis (whereby the lower values in the grey area refer to low fitness and the high ones to high fitness values).

The joint influence of antiviral prophylaxis and fitness of the resistant strain is shown in Figs. 3a–c. We assume that during the whole course of the epidemic 0 to 20% of people between 20 to 60 years of age receive prophylaxis. Fitness of the resistant virus is varied from 80 to 100% (simulations with lower fitness values yield results which are very similar to the 80% scenario). Antiviral prophylaxis increases the number of treatment failures in the population (Fig. 3a). If the fitness of the resistant strain is less than 80%, this effect may be regarded as negligible, but for higher fitness values and notably for a combination of high fitness and large prophylaxis coverage, the results become increasingly pessimistic. If the fitness of the resistant strain is below 88%, a low coverage of antiviral prophylaxis can lead to a slightly smaller number of hospitalizations, whereas for larger fitness values, the expected number of hospitalizations grows with growing prophylaxis coverage. In the case of 100% fitness, the expected number of hospitalizations grows from 314 per 100,000 (without prophylaxis) to 516 (20% receive prophylaxis). Apart from hoping that the population may benefit from antiviral prophylaxis via herd effects, the main goal of giving prophylaxis may be to maintain the work force of first responders. The expected work loss per person can approximately be calculated as follows: omitting antiviral treatment and prophylaxis, we expect about 75% of the population to become infected. One third of the infected individuals becomes severely sick for an average of 7 days and needs a further 5 days to recover. In total, 25% of all working adults should, therefore, be out of work for 12 days, i.e. we expect an average sickness related work loss of 3 days per person in this scenario. This value is further reduced by therapeutic antiviral treatment, partly because treated individuals recover more quickly, but more importantly because treatment prevents the transmission of infection and thereby has a beneficial herd effect. If all severe cases are treated, the expected work loss per person drops to 2.1 days if no NI resistance develops or if the NI resistant strain has a low fitness (with *de novo *development of a highly transmissible resistant strain, the expected work loss is only reduced to 2.4 days). As a reference value, the expected work loss per person without prophylaxis is depicted as grey horizontal bar in Fig. 3c. Values below the grey bar indicate that people who receive prophylaxis have a benefit, values above indicate that their work loss is higher than what would have been expected if nobody had received prophylaxis. If only few people receive prophylaxis, their work loss can be less than half of the value without prophylaxis, especially if the fitness of the resistant strain is low. For strains with a fitness of less than 92%, up to 20% may benefit from prophylaxis. For strains with higher fitness, the expected work loss can be worse than without prophylaxis if 10% or more receive prophylaxis.

### Uncertainty analyses

In the following, we investigate the influence of parameter uncertainty by systematically varying the values of two unknown parameters:

(a) The susceptibility to infection *x*_*sus *_of people who take prophylaxis is varied from 0 to 100% (baseline value 50%). The results only deviate from the values shown in Figs. 3a–b by up to 1240 treatment failures and by up to 21 hospitalizations per 100,000 inhabitants (with a slight tendency towards higher deviations for combinations of high prophylaxis coverage and high fitness values). The parameter *x*_*sus *_strongly influences the expected work loss of people who take prophylaxis. If only few people take prophylaxis (cf. Fig. 3c), the expected work loss varies between nearly 0 days (*x*_*sus *_= 0) and 1.2 days (*x*_*sus *_= 1) for low fitness values, and from 0.9 to 1.6 days for high fitness values. If 10% of the population between 20 and 60 years of age take prophylaxis, the expected work loss varies from 0.1 day (*x*_*sus *_= 0) and 1.1 days (*x*_*sus *_= 1) for low fitness values, and it is about 2.2 days (irrespective of *x*_*sus*_) for high fitness values.

(b) The fraction *x*_*imm *_of asymptomatic cases who develop protective immunity if they have received prophylaxis, is varied from 0 to 100% (baseline value 50%). The results only deviate from the values shown in Figs. 3a–c by up to 240 treatment failures, by up to 7 hospitalizations and by up to 0.1 days of work loss per person.

### Quick calculation formula of the critical fitness of the resistant strain

The basic reproduction number *R*_0 _is the expected number of people who are infected by a single index case in a fully susceptible population where no interventions are taken. If part of the population is immune and if interventions are taken, we talk about the effective reproduction number: the effective reproduction number $R_{e}^{sens}$ of the drug sensitive strain and that of the NI resistant strain ($R_{e}^{res}$) can be approximated as follows:

$$\begin{array}{l} R_{e}^{sens}\approx R_{0}s(1-r_{SD})(1-r_{iso})(1-r_{tr})(1-r_{pro})\\ R_{e}^{res}\approx R_{0}s(1-r_{SD})(1-r_{iso})f \end{array}$$

Here, *s *is the susceptible fraction of the population, *r*_*SD *_is the reduction of transmission due to social distancing, *r*_*iso *_is the reduction due to case isolation, *r*_*tr *_is the reduction of transmission due to antiviral treatment of cases, *r*_*pro *_is the fraction of the population protected by prophylaxis and *f *is the fitness of the resistant strain (for more details, see the additional file SupplementaryData.doc). An infection can only be transmitted if the effective reproduction number is larger than 1. For the NI resistant strain, $R_{e}^{res}$ is larger than 1 if the fitness *f *is larger than (*R*_0_*s *(1- *r*_*SD*_)(1 - *r*_*iso*_))^-1^. In a fully susceptible population (*s *= 1) where no interventions are performed (*r*_*SD *_= *r*_*iso *_= 0), the fitness *f *must be larger than $R_{0}^{-1}$ = 40%. Immunity in the population and contact reductions increase the critical value of *f*. NI resistance only becomes a problem if the resistant strain spreads more efficiently than the drug sensitive one, i.e. if $R_{e}^{res}>R_{e}^{sens}$. This is the case if *f *> (1 - *r*_*tr*_)(1 - *r*_*pro*_). Note that the critical value of *f *is independent of contact reduction measures; it only depends on the effects of treatment and prophylaxis. If the overall treatment effect is *r*_*tr *_= 0.188 (see additional file SupplementaryData.doc), the critical fitness of the resistant strain is about 81% if no prophylaxis is given. If the fitness exceeds this value, the resistant strain will invariably take over. The critical value for the fitness drops to 73% and 65%, respectively, if 10% or 20% of the population receive prophylaxis.

## Discussion

Although NI resistant viruses may emerge *de novo *in treated patients in such a late state of their course of disease that most patients may not pass on the infection, our simulation study shows that the resistant strain will become highly prevalent in the population if its relative fitness is high and if NI treatment or prophylaxis are common. Prior to the 2007/8 influenza season, NI resistant strains were only infrequently found in patients after treatment with oseltamivir and in patients not exposed to oseltamivir. Early surveillance data from the 2007/8 influenza season on the northern hemisphere suggest that an oseltamivir resistant influenza virus type A (H1N1) circulates in several European countries and in North America [6,7,11]. Resistance infections have been reported from over two third of the countries which have implemented an influenza surveillance system and test for antiviral resistance. Furthermore, the proportion of resistant infections has become alarmingly high (between 4% and 70%) in the afflicted countries. Even in a fully susceptible population, a resistant virus can only spread if its fitness exceeded 40%. The populations in which the resistant seasonal influenza virus is spreading are far from being susceptible which further increases the minimum fitness of the resistant strain.

Considering the growing prevalence of resistant infections in spite of extremely low treatment rates, the fitness of the current resistant strain must indeed be very high. It is conceivable that a pandemic influenza strain may also become resistant without a considerable loss of transmissibility although this may be regarded as a worst case scenario. In a pandemic scenario, NI treatment is one of the major means of intervention and will be used extensively. Containing a potential pandemic within the country of its origin by widespread antiviral prophylaxis has been suggested [1,3]. Prophylaxis has also been considered for local interventions after the international spread of the pandemic virus.

**Figure 4:**
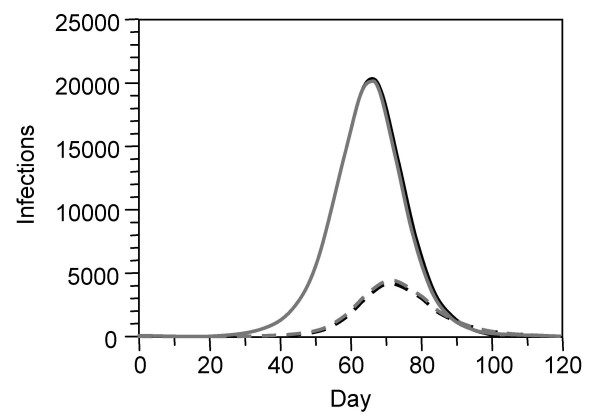
**Comparison of *de novo *development of resistance and importation of resistance**. Prevalence of infection with drug sensitive (solid curves) and drug resistant infection (dashed curves) during a pandemic wave in a population of 100,000 inhabitants. Scenario (a): a drug-sensitive infection is introduced on day 0; NI resistant infection develops *de novo *during antiviral treatment of cases throughout the simulation (treatment parameters see Figure 2; no prophylaxis; 100% fitness of the resistant virus; black curves). Scenario (b): the importation of a drug sensitive infection on day 0 is followed by an importation of a NI resistant infection on day 28. Treatment is given as in scenario (a), but no *de novo *development of resistance occurs within this scenario (grey curves). The resulting curves for Scenario (a) and (b) are nearly indistinguishable.

Even in the optimistic scenario where a drug sensitive infection is introduced into a population, *de novo *development of NI resistance in treated patients and the ensuing spread of resistant infections may lead to an early predominance of a resistant strain (Figs. 2a–c). *De novo *development of resistance in a person is a stochastic event and would demand for stochastic simulation in order to realistically describe the variability in the timing of such an event. Deterministic models like *InfluSim *only represent the average course the development of resistance in a population. For sake of simplicity, we have assumed that the development of resistance occurs in one step whereas other authors [18] assume that the first mutation leads to a resistant virus with impaired fitness and that the transmission fitness will gradually improve over time. Once a resistant virus with high fitness spreads in the population, the relatively rare *de novo *development of resistance in other people can be completely neglected because it is out-weighted by the multiplication of the virus in the population. This is because therapeutic and prophylactic NI use put pressure on the drug sensitive strain and favor the spread of circulating NI resistant infections. Figure 4 demonstrates this by comparing two scenarios: In scenario (a), NI resistance develops *de novo *by treatment of cases whereas in scenario (b) a NI resistant infection is introduced 4 weeks later, but no *de novo *development of NI resistance occurs. The resulting curves are nearly indistinguishable, indicating that once a resistant strain of high transmissibility spreads in a population where there is a lot of pressure on drug sensitive infection, any further *de novo *development of resistance can be neglected. Our calculations show that under widespread treatment, the NI resistant strain spreads faster than the non-resistant one if its fitness exceeds 81%. Prophylaxis will further increase the pressure, leading to a quicker replacement of the drug sensitive strain by the resistant one and increasing the number of unsuccessfully treated patients (Fig. 3a). If the fitness of the resistant virus is between 90 and 100%, prophylaxis even increases the total number of cases (Figs. 2a–c) and hospitalizations (Fig. 3b), and we obtain the counter-intuitive result that the work loss of those people who receive prophylaxis may become larger than without prophylaxis (Fig. 3c). Our simulations assume that a small fraction of the population receives prophylaxis during the whole course of the epidemic whereas individuals are only advised to take prophylaxis for a maximum of six weeks [19]. Another approach would be to split up the group of first responders into subgroups who alternatively receive prophylaxis, but the pressure on the drug sensitive virus exerted by prophylaxis mainly depends on the prophylaxis coverage and does not change much if different people receive the drug at different times. Especially for low prophylaxis coverage, our results should be relatively robust in spite of this over-simplification. Our simulation results confirm other authors' findings which indicate that the benefits of antiviral drug use to control pandemic influenza may be reduced by NI resistance in the virus [5,6]. Our model structure, assumptions and parameter values differ from those reports: we used higher values for *R*_0 _and for *de novo *development of NI resistance than [5], but lower values than [6], and we used a wide range of prophylaxis levels. In contrast to [5] and [6], we found clear detrimental effects of any level of prophylaxis if the relative fitness of the resistant strain is higher than 80% [20,21].

## Conclusion

Surveillance of the appearance of NI resistant infections and, most importantly, of the fitness of resistant strains will be crucial to manage a pandemic wave. Uncontrolled use of NI may do more harm than good. If a NI resistant pandemic strain is reported to spread, the use of NI should mainly be restricted to the treatment of cases whereas prophylaxis must be reduced to an absolute minimum. If the fitness of the NI resistant pandemic strain is high, any use of prophylaxis may increase the number of hospitalizations and deaths in the population. Thus, public health systems should diversify intervention strategies by using prophylaxis in a very restrictive manner, by stockpiling different types of antiviral drugs and by supplementing pharmaceutical interventions with social distancing measures [22,23].

## Competing interests

The authors declare that they have no competing interests.

## Authors' contributions

ME was responsible for model design, mathematical formulas and the writing of manuscript. MS designed and programmed the software and provided the simulation results. HPD advised on modeling questions, helped preparing the figures and proofread the manuscript. MW gave epidemiologic advice and co-wrote the manuscript. DK and SOB defined the research questions, made public health decisions and proofread the manuscript. BV coordinated the research project and co-wrote the manuscript. All authors have read the manuscript and have given final approval of the current version to be published.

## Pre-publication history

The pre-publication history for this paper can be accessed here:

http://www.biomedcentral.com/1471-2334/9/4/prepub

## Supplementary Material

Additional file 1 Age-dependent parameters and effective reproduction number. Click here for file
