# Supplementary material for: Antiviral prophylaxis during pandemic influenza may increase drug resistance
Source: BMC Infect Dis. 2009 Jan 20;9:4. doi: 10.1186/1471-2334-9-4 (PMC2654456; doi:10.1186/1471-2334-9-4)
Supplement: Additional file 1 — Age-dependent parameters and effective reproduction number. [file 1471-2334-9-4-S1.doc]

Supplementary Data

Age-dependent parameters

Table A1. Age distribution of the Swiss population (per 100,000), contact matrix and risk of hospitalization and death from influenza by age class and risk group.

|  | Children | Working adults | Elderly |
| --- | --- | --- | --- |

| Age in years | 0-5 | | 6-12 | | 13-19 | | 20-39 | 40-59 | 60 or more | |
| --- | --- | --- | --- | --- | --- | --- | --- | --- | --- | --- |
| Number per 100,000^1^ | 5,895 | | 7,701 | | 8,315 | | 27,318 | 29,121 | 21,650 | |
| Contacts per week^2^ |  | | | | | |  | |  | |
| with 0-5 year old | 169.14 | 31.47 | | | | 17.76 | 34.5 | 15.83 | 11.47 | |
| with 6-12 year old | 31.47 | 274.51 | | | | 32.31 | 34.86 | 20.61 | 11.50 | |
| with 13-19 year old | 17.76 | 32.31 | | | | 224.25 | 50.75 | 37.52 | 14.96 | |
| with 20-39 year old | 34.5 | 34.86 | | | | 50.75 | 75.66 | 49.45 | 25.08 | |
| with 40-59 year old | 15.83 | 20.61 | | | | 37.52 | 49.45 | 61.26 | 32.99 | |
| with $\geq$ 60 year old | 11.47 | 11.50 | | | | 14.96 | 25.08 | 32.99 | 54.23 | |
|  |  | | | | | |  | |  | |
| Risk category | low risk | | | high risk | | | low risk | high risk | low risk | high risk |
| Fraction of age class^3^ | 90% | | | 10% | | | 85% | 15% | 60% | 40% |
| Fraction of infected who become severely sick*^4^ | 33% | | | 33% | | | 33% | 33% | 33% | 33% |
| Fraction of severely sick who need hospitalization*^3^ | 0.187% | | | 1.333% | | | 2.339% | 2.762% | 3.56% | 7.768% |
| Fraction of hospitalized patients who die^5^ | 5.541% | | | | | | 16.531% | | 39.505% | |

*) The fractions of severely sick and hospitalized patients assume that the patients neither receive NI treatment nor prophylaxis, or that they are infected with a drug resistant virus.

^1^) Swiss Federal Statistical Office, Swiss population 2005

^2^) Wallinga J, Teunis P, Kretzschmar M. Using social contact data to estimate age-specific transmission parameters for infectious respiratory spread agents. *Am J Epidemiol* 2006; 164: 936-44.

^3^) Influenzapandemieplanung: Nationaler Deutscher Influenzapandemieplan. *Bundesgesundheitsblatt - Gesundheitsforschung - Gesundheitsschutz* 2005; 48: 356-90.

^4^) Longini IM, Jr., Halloran ME, Nizam A, et al. Containing pandemic influenza with antiviral agents. *Am J Epidemiol* 2004; 159: 623-33.

^5^) Meltzer MI, Cox NJ, Fukuda K. The economic impact of pandemic influenza in the United States: priorities for intervention. *Emerg Infect Dis* 1999; 5: 659-71.

Effective reproduction number

The basic reproduction number $R_{0}$ is the expected number of secondary infections caused by one infected individual during the whole course of the infectious period if no interventions are performed and if the whole population is susceptible. As the simulation program *InfluSim* uses a contact matrix (Table A1), the expected numbers of secondary infections differ depending on the age of the infected person and of the contact. Furthermore, asymptomatic individuals are only half as contagious as symptomatic ones and can also have a shorter infectious period (depending on age). In order to calculate a simplified rule of thumb formula, we will use the overall average of $R_{0}$ secondary infections and neglect interactions between different interventions.

If part of the population is immune and if interventions are performed, the basic reproduction number is reduced to what we call the effective reproduction number $R_{e}$: If only a fraction $s$ of the population is susceptible, we obtain $R_{e}=sR_{0}$. If the population prevents a fraction $r_{SD}=0.1$ of all contacts, the effective reproduction number further reduces to $R_{e}=sR_{0}(1-r_{SD})$.

Partial isolation reduces the effective reproduction number by a factor $(1-r_{iso})$. In this paper, we assume that moderately sick patients reduce their contacts by 10%, severely sick patients who are taken care of at home by 20% and hospitalized patients by 30%; individuals with asymptomatic infection do not further reduce their contacts. As hospitalized cases are relatively rare, their slightly higher isolation effect (30% reduction) can be neglected. This leaves us with one third asymptomatic infections, one third moderately sick and one third severely sick cases (Longini 2004). As asymptomatic individuals are assumed to be only half as contagious as the others (Longini 2004), the total contagiousness is distributed among the three types of cases (asymptomatic, moderately sick and severely sick) as
 $\frac{0.6}{3}+\frac{1.2}{3}+\frac{1.2}{3}=100\%$ and we can approximately calculate the isolation effect as
$r_{iso}\approx\frac{0.6}{3}*0+\frac{1.2}{3}*0.1+\frac{1.2}{3}*0.2=12\%$.

Treatment of cases reduces the effective reproduction number by a factor $(1-r_{tr})$. Only the one third of cases that suffers severe disease seeks medical help and receives antiviral treatment. Our assumption that the infectiousness drops exponentially over the course of the disease and that 90% of the contagiousness of each case are concentrated within the first half of the infectious period, leads to a relative contagiousness $f(t)$ which decreases over disease time $t$ according to $f(t)=0.636*e^{-0.628 t}$. On average, cases visit a medical doctor one day after onset of symptoms (i.e. when they have already spent 44.7% of their contagiousness), so that at most 55.3% of their contagiousness can be prevented by treatment. NI treatment reduces their contagiousness by 80% (Longini 2004) and their remaining duration of disease by 25%. Combining all these values, we get approximately $r_{tr}\approx\frac{1.2}{3}*0.553*(1-(1-0.8)(1-0.25))=18.8\%$, i.e. a little more than half the contagiousness of one third of all infected individuals can be prevented by treatment.

In our quick calculation formula, prophylaxis reduces the effective reproduction number by a factor $(1-r_{pro})$. This again is a simplification (the exact treatment of this problem would demand to separate the population into groups with and without prophylaxis and to separately calculate the effects of prophylaxis on the susceptibility and contagiousness of these individuals, turning the calculation of $R_{e}$ into a rather complicated eigenvalue calculation). In our simulations, prophylactic treatment is assumed to reduce the susceptibility of individuals by 50%. Upon infection, it reduces their contagiousness (like treatment) by 80% and the duration of sickness by 25%. It also leads more frequently to asymptomatic infection (i.e. a state with lower contagiousness), but may prevent some of the asymptomatically infected individuals from becoming immune. For the rule of thumb formula, we make the simplifying assumption, that people who take prophylactic treatment are fully prevented of either acquiring or passing on the infection (combining the values given above leads to an effect of over 90% instead of the simplified 100%).

Combining all these assumptions, the effective reproduction number of the drug sensitive strain is $R_{e}^{sens}\approx R_{0}s(1-r_{SD})(1-r_{iso})(1-r_{tr})(1-r_{pro})$.

The drug resistant virus does not respond to NI treatment, but may have a reduced fitness $f$. Thus, its effective reproductive number simplifies to

$R_{e}^{res}\approx R_{0}s(1-r_{SD})(1-r_{iso})f$.
